# Incidence, causes and factors associated with torso injury

**DOI:** 10.7196/AJTCCM.2021.v27i3.169

**Published:** 2021-10-04

**Authors:** Ivan Schewitz

**Affiliations:** Department of Cardiothoracic Surgery, University of Pretoria, South Africa

## Editorial

Road traffic accidents are the most common cause of torso injuries worldwide. The article by Wepngong *et al*. ^[1]^ in this issue of the AJTCCM stresses the effect this has on the morbidity and mortality and the long-term effects on patients. This is a timely reminder of the effects of this pandemic and the long-term effects on communities.

On average, the COVID-19 pandemic causes 2 205 deaths per day worldwide.^[2]^ This is in comparison with 3 406 deaths from road accidents, 3 243 from tuberculosis, 3 753 from diabetes, 2 615 from HIV/AIDS and 2 175 from suicide. Road accident deaths is a greater pandemic worldwide than COVID-19. It is something that we have had in South Africa for generations and that costs the country a great deal economically and is not headline news. If our reaction to the road accident pandemic was similar to what has occurred with COVID-19, we would ban interprovincial travel, close all liquor outlets permanently, have a nightly curfew from 21h00 to 04h00 and prevent gatherings of >50 people, especially major events such as political rallies and soccer and rugby tournaments.

The most common cause of torso injuries, as shown by Wepngong *et al*.,^[1]^ are road accidents with a significant number of the injured needing time off work. In low- (LIC) and middle-income countries (MIC) such as we have in many African countries, this is of major significance. Most of these patients are young and the effect on the economy is significant.

Wepngong *et al*. ^[1]^ mention the use of informal healthcare professionals and the effect this has on late presentation of severe injuries associated with the torso.

So what can we do to decrease road accident trauma? We cannot really respond in the way we have responded to the COVID-19 crisis, can we? The five main risk factors as shown by Híjar *et al*. ^[3]^ are speed management, drinking and driving, motorcycle helmet use, seatbelt use, and use of child restraints; however, without adequate legislation and enforcement of the rules, these factors are of no use. In Africa, we have the legislation but very often there is no enforcement of the law. In the Americas, Híjar *et al*. ^[3]^ showed that legislation in most countries is more than adequate but enforcement is inadequate in many cases. An example is speeding, and drinking and driving, where legislation is 100% but enforcement at 10% or less.^[3]^ Urie *et al*. ^[4]^ have shown that as we progress from high- to low-income countries, the compliance with legislation decreases. With this comes increasing road accidents and trauma. Urie *et al*. ^[4]^ conclude that ‘efficient law enforcement and effective safety education taking into account cultural diversity are the key aspects to reduce traffic-related injuries and fatalities in low-income countries like India’ and, I would add, many countries in Africa.

Forjuoh^[5]^ noted the difficulty governments in developing countries face in enforcing legislation, and mentioned simple measures that can be introduced to reduce road accident trauma. These include enforcing legislation, roadway barriers and selected traffic-calming designs, pedestrian crossings and public education. Costs are always a problem in LICs, as are competing healthcare issues. LICs may have to improvise and innovate in traffic safety. Rumble strips and speed humps were found to be effective on Ghanaian roads.^[6]^

Increased road accident morbidity and mortality in socioeconomically disadvantaged communities in LICs mirror the health inequities in the country. We can begin to address these with increased education and, very importantly, by enforcing the legislation which we already have. The contribution by Wepngong *et al*. ^[1]^ highlights an important and ongoing pandemic.
